# Promoting Factors for Physical Activity in Children with Asthma Explored through Concept Mapping

**DOI:** 10.3390/ijerph16224467

**Published:** 2019-11-13

**Authors:** Annette Brons, Katja Braam, Annieck Timmerman, Aline Broekema, Bart Visser, Bart van Ewijk, Suzanne Terheggen-Lagro, Niels Rutjes, Hellen van Leersum, Raoul Engelbert, Ben Kröse, Mai Chinapaw, Teatske Altenburg

**Affiliations:** 1 Digital Life Center, University of Applied Sciences Amsterdam, Wibautstraat 2, 1091 GM Amsterdam, The Netherlands; b.j.a.krose@hva.nl; 2 ACHIEVE, University of Applied Sciences Amsterdam, Tafelbergweg 51, 1105 BD Amsterdam, The Netherlands; k.i.braam@hva.nl (K.B.); anniecktimmerman@gmail.com (A.T.); a.broekema@hva.nl (A.B.); b.visser2@hva.nl (B.V.); r.h.h.engelbert@hva.nl (R.E.); 3 Department of Pediatric Pulmonology, Tergooi Hilversum, van Riebeeckweg 212, 1213 XZ Hilversum, The Netherlands; bvanewijk@tergooi.nl; 4 Department of Pediatric Pulmonology, Academic Medical Center Amsterdam, Meibergdreef 9, 1105 AZ Amsterdam, The Netherlands; s.w.terheggenlagro@amsterdamumc.nl (S.T.-L.); n.w.rutjes@amsterdamumc.nl (N.R.); 5 Department of Pediatrics, Kinderkliniek Almere, Hospitaaldreef 29, 1315 RB Almere, The Netherlands; hvleersum@dekinderkliniek.nl; 6 Department of Rehabilitation, Academic Medical Center Amsterdam, Meibergdreef 9, 1105 AZ Amsterdam, The Netherlands; 7 Informatics Institute, University of Amsterdam, Science Park 904, 1098 XH Amsterdam, The Netherlands; 8 Department of Public and Occupational Health, Amsterdam Public Health Research Institute, Amsterdam UMC, Vrije Universiteit Amsterdam, Van der Boechorststraat 7, 1081 BT Amsterdam, The Netherlands; m.chinapaw@amsterdamumc.nl (M.C.); t.altenburg@amsterdamumc.nl (T.A.)

**Keywords:** concept mapping, physical activity, exercise, asthma, children, parents, healthcare providers

## Abstract

For children with asthma, physical activity (PA) can decrease the impact of their asthma. Thus far, effective PA promoting interventions for this group are lacking. To develop an intervention, the current study aimed to identify perspectives on physical activity of children with asthma, their parents, and healthcare providers. Children with asthma between 8 and 12 years old (*n* = 25), their parents (*n* = 17), and healthcare providers (*n* = 21) participated in a concept mapping study. Participants generated ideas that would help children with asthma to become more physically active. They sorted all ideas and rated their importance on influencing PA. Clusters were created with multidimensional scaling and cluster analysis. The researchers labelled the clusters as either environmental or personal factors using the Physical Activity for people with a Disability model. In total, 26 unique clusters were generated, of which 17 were labelled as environmental factors and 9 as personal factors. Important factors that promote physical activity in children with asthma according to all participating groups are asthma control, stimulating environments and relatives, and adapted facilities suiting the child’s needs. These factors, supported by the future users, enable developing an intervention that helps healthcare providers to promote PA in children with asthma.

## 1. Introduction

Physical activity (PA) is beneficial for children’s health [1]. This especially holds for children who suffer from a chronic disease [2,3]. Asthma is the most frequently diagnosed chronic disease among children [4]. In The Netherlands, approximately 1 out of 15 children is diagnosed with asthma [5]. Asthma is a chronic inflammatory disorder of the airways, characterised by recurrent attacks of breathlessness and wheezing [4]. These symptoms can be provoked by triggers such as allergens, smoke, and weather conditions, as well as by PA. While the severity and frequency of the attacks vary among patients, the majority of the population can control their asthma with the use of medication and avoiding triggers.

**Figure 1:**
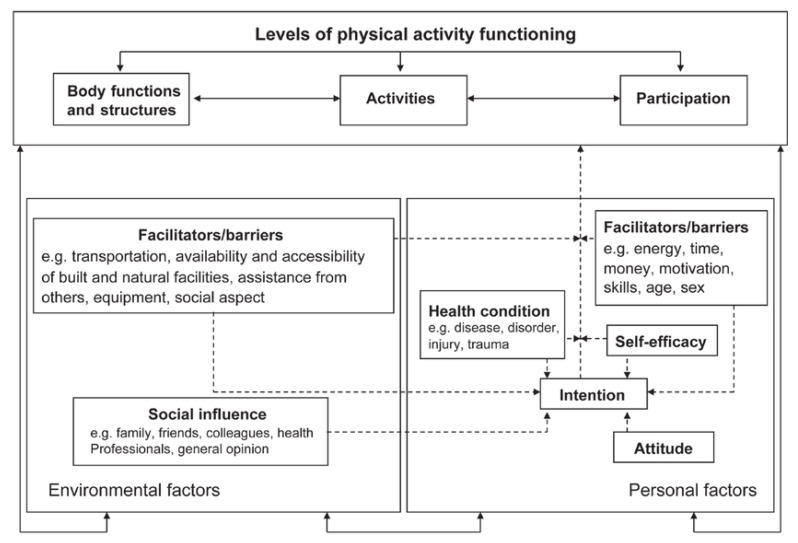
Physical Activity for People with a Disability (PAD) model. Reprinted from “Physical activity for people with a disability” by H.P. van der Ploeg, 2004, *Sports Medicine*, 34(10), p. 645. Reprinted with permission from the author.

Although PA can trigger asthma symptoms, it can also positively affect children’s asthma control by improving their physical fitness. Better asthma control leads to higher quality of life, decreased medication use and reduced hospitalisation [2,6,7,8]. Despite the positive effects of PA on physical fitness and asthma control, children with asthma, especially girls, seem to engage less in PA than their healthy peers [9,10,11]. Asthma specific underlying causes of decreased PA can be dyspnea, fear of dyspnea, and dysfunctional breathing [12]. A review on factors associated with participation in PA in children with disabilities or chronic diseases showed that underlying causes of decreased PA are also related to low self-esteem, lack in motivation, and inadequate physical fitness [13]. According to the Physical Activity for people with a Disability (PAD) model [14], all factors impacting on PA can be categorised as either personal or environmental factors. As can be seen in Figure 1, personal factors include intention, attitude, self-efficacy, health condition, and personal barriers and facilitators while environmental factors include social influence, and environmental barriers and facilitators.

Children with asthma who engage only a small amount of time in PA are referred to healthcare providers, such as paediatric physical therapists and pulmonary nurse practitioners. There they participate in exercise training, learn how to cope with exercise barriers, and are motivated to be more physically active. Healthcare providers indicate that they need interventions that are effective in improving and maintaining PA for children with asthma on the long term. To develop a potentially effective intervention, it is important to implement promoting factors that suit the needs of children with asthma. In the current literature, these factors are mostly studied from the perspective of parents and experts instead of children and the focus is often on barriers instead of facilitators [15,16]. Thus, better insight in promoting factors from the perspective of children with asthma is needed. Furthermore, studying the factors from the perspective of parents and healthcare providers is also important since parents know their children best and play an important role in the PA behaviour of their child, and healthcare providers play an important role in promoting PA in these children and controlling their asthma.

Better insight into the factors that promote their PA is required to develop an intervention that increases PA levels in children with asthma. Here, increasing PA levels is about increasing PA in general and not specifically about sports activities. In the process of developing an intervention that fits the needs of these children, insight into these factors is required. Therefore, three stakeholder groups are important: children with asthma themselves, who are the experts of their own behaviour; parents of children with asthma, who know how to stimulate their children; and healthcare providers of children with asthma, who have to implement the intervention in their care and have important knowledge about the medical barriers. Accordingly, the aim of our study is to explore the promoting factors of PA in children with asthma through the perspective of children with asthma, their parents, and their healthcare providers to implement these factors in the intervention.

Concept mapping (CM) is a helpful participatory method to acquire knowledge from a participant’s point of view. It combines qualitative and quantitative methods by collecting data in a structured way and analysing this data quantitatively [17,18]. To implement the most important factors in an intervention, the aim of our study is to explore the promoting factors of PA in children with asthma from the perspective of children with asthma, their parents, and their healthcare providers.

## 2. Materials and Methods

### 2.1. Recruitment

Children and their parents were recruited through paediatric pulmonologists and paediatric physiotherapists in three different hospitals in The Netherlands specialised in paediatric asthma (Amsterdam University Medical Center, Amsterdam; Tergooi Hospital, Hilversum; and Kinderkliniek, Almere). Children who were diagnosed with asthma, aged between 8 and 12 years old, and either active or inactive could be included. Children who met the inclusion criteria received a study information letter from their physician. Their physician reported their age, activity level, and asthma status. The activity level was based on their PA participation in a general week and was labelled by their physician as low (not doing sports at all and not playing outside regularly), moderate (playing sports once a week and playing outside regularly), and high (playing sports at least twice a week and playing outside regularly) levels of PA. The asthma status, which corresponds to the level of asthma control, was defined as mild/well controlled, moderate/partially controlled, or severe/poorly controlled. All children are treated by a paediatric pulmonologist in a specialised hospital who prescribes medication according to the “Dutch Paediatric Society” guidelines for asthma in children [19]. The treatment includes taking medication before PA to prevent or diminish exercise induced symptoms.

Written informed consent was obtained from both parents or legal guardians for participation of a child. A separate individual written informed consent was acquired for participation of a parent. In total, 28 children and 23 parents gave written informed consent and, respectively, 25 and 17 actually participated.

Healthcare providers could be included if they were either physical therapist or lung nurse practitioner and if they had experience in treating children with asthma. Information letters were sent to potential participants via both a Dutch paediatric physical therapist profession group and the paediatricians of the participating hospitals. A written informed consent was obtained for participation. In total, 23 healthcare providers gave consent and started the study; two dropped out due to illness.

The AMC Medical Ethical Committee approved the research protocol with trial number METC 2017 191. This study was registered in the Dutch Trial Registry with trial number NTR6658 on 21 August 2017.

### 2.2. Procedures

**Figure 2:**
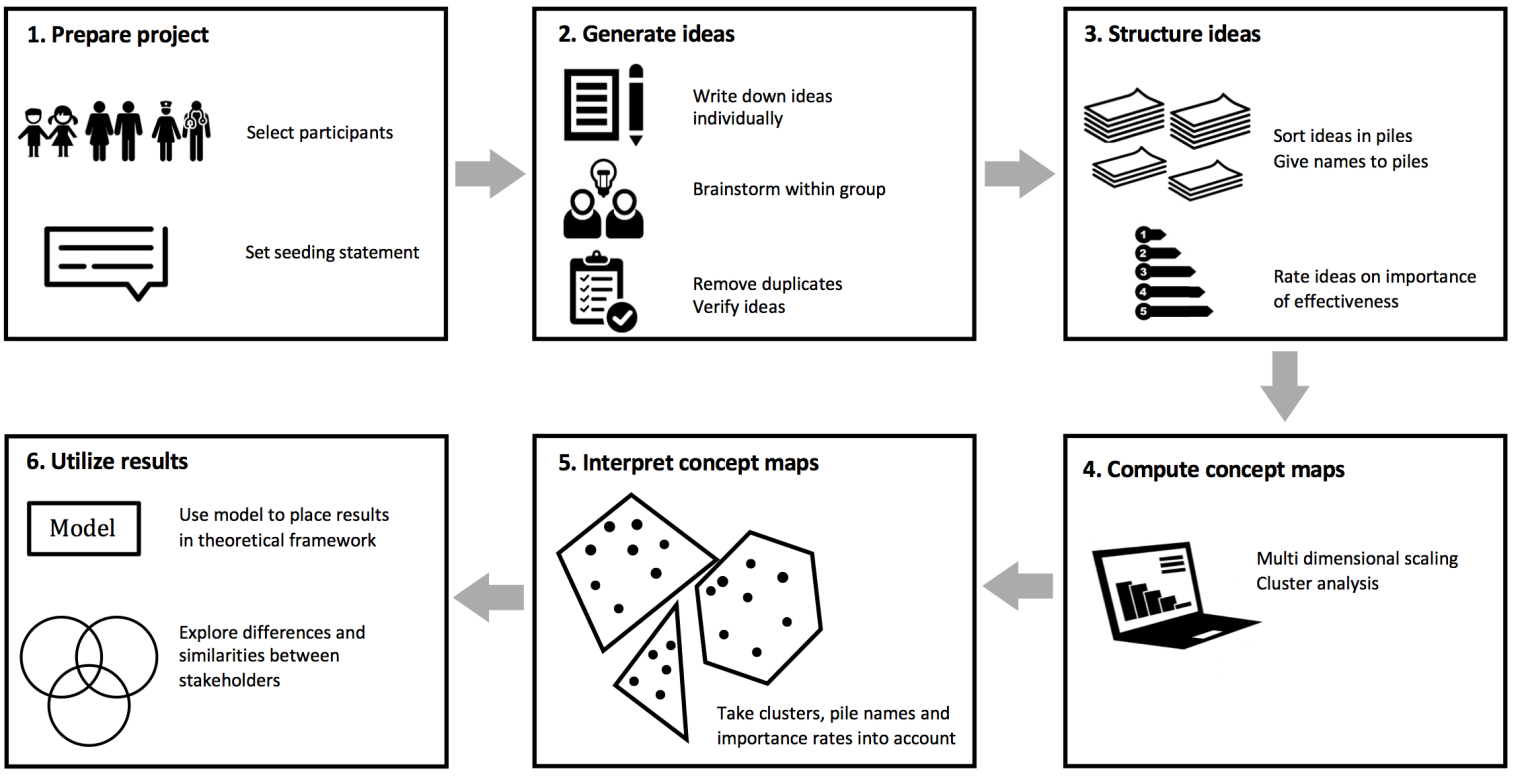
Overview of the six steps of the concept mapping method.

The CM method consists of six steps, which are shown in Figure 2. Data were collected between November 2017 and March 2018. Descriptive participant characteristics, means and standard deviations were calculated with R version 3.4.1.

#### 2.2.1. Concept Mapping Sessions with Children and Parents

The CM sessions for the children and parents were organised on two different dates. Sixteen children, divided into two groups of seven and nine children, and twelve parents participated on the first day. Nine children, divided into two groups of four and five children, and five parents participated on the second day. This resulted in a total of four groups of children and two groups of parents with whom CM sessions were held. All groups were facilitated by one or two trained researchers. Each researcher had received a total of eight hours of training, consisting of reading the concept mapping manual and attending an oral presentation provided by an expert in concept mapping. Thereafter, a pilot test of the concept mapping session was performed with a group of five healthy children. The final concept mapping sessions took approximately five hours and were split into two parts with a fun and interactive workshop in between.

To get familiar with the CM method and the subject of asthma and PA, all groups started with a warming up question: “Which activities belong to the definition of physical activity?”. Thereafter, the seeding statement was presented in two ways: “What would help a child with asthma to become more physically active” and “A child with asthma would be more physically active if ...”. Each participant individually wrote down as many ideas they could think of in response to the seeding statement. Subsequently, the participants shared their ideas one by one in a group brainstorm, during which participants could come up with new ideas as well. The ideas were checked on uniqueness and clarity by the group, resulting in a list of unique ideas.

The unique ideas were subsequently printed on separate cards for each participant. Each participant individually grouped all ideas into piles based on similarity, and named all piles. A minimum of two piles was required with each pile containing at least two ideas. An “other” or “miscellaneous” pile was prohibited. Subsequently, each participant rated the importance of all ideas in being helpful to become more physically active, using a five-point Likert scale ranging from very unimportant (1) to very important (5).

#### 2.2.2. Concept Mapping Sessions with Healthcare Providers

CM sessions with healthcare providers were performed online with a self-developed web application. The process of the CM session was comparable to the session of the children and parents, except for the group brainstorm since the professionals individually used the online web application. After the researchers deleted duplicates, each participant received the list of all unique ideas from all healthcare providers together. Each participant was asked to check the list on the clarity of each idea. They were stimulated to add new ideas when they were inspired by other ideas on the list. Based on their comments and additions, the researchers generated the final list of unique ideas that was presented in the web application. Next, participants were asked to perform the sorting, naming and rating tasks using the web application.

#### 2.2.3. Generating the Concept Maps

For each participant of a group, a matrix was built based on the piles they composed. In this, a cell (i,j) contained 1 in case the participant stored idea *i* and *j* in the same pile, and 0 if the ideas were placed in different piles. Then, the matrices of all participants in the same group (i.e., all matrices of the participants that sorted the same list of unique ideas) were summed. Subsequently, the result was divided by the number of participants. This resulted in a $k_{g}\times k_{g}$ distance matrix per group, with $k_{g}$ being the number of ideas within a group, in which all pairwise distances between all ideas were represented. Since it is impossible to visualise a *k*-dimensional space, the distance matrix had to be transformed into a two-dimensional space. This was done using the multidimensional scaling (MDS) algorithm [20]. The stress factor indicates how well the original pairwise distances in the *k*-dimensional space are preserved in the two-dimensional space. The MDS algorithm optimises this stress factor resulting in a two-dimensional space in which the original pairwise distances are represented to the best extent possible. Subsequently, the relative distances in the two-dimensional space were visualised. In this representation, ideas that participants often placed in the same pile appear closer together than ideas that were less often grouped together. Clusters of ideas were made with hierarchical cluster analysis. Specifically, Ward’s method, which minimises the within-cluster variance, was used. The default number of generated clusters was 6. The average importance values for each cluster was calculated based on the importance rating of the underlying ideas.

RCMap was applied to create the concept maps from the sorted ideas of the participants [21]. This open source software is implemented for R, of which we used version 3.4.1.

#### 2.2.4. Interpreting the Concept Maps

Four researchers (A.B., K.B., A.T., and A.Br.) independently explored the clusters on interpretation and analysed whether more or fewer clusters would represent the participant’s ideas better. The final clusters were named by the researchers based on the pile names of all participants.

To place all clusters in a theoretical framework, two researchers (A.B., K.B.) categorised all clusters of all concept maps according to the categories of the PAD model. A cluster was labelled as either a personal factor (attitude, intention, self-efficacy, health condition, or personal facilitators and barriers) or an environmental factor (social influence, or environmental facilitators and barriers). To visualise the overlap and differences between all stakeholder groups, we represented the unique clusters in a Venn diagram.

## 3. Results

### 3.1. Participant Characteristics

The 25 participating children were on average 9.6 ± 1.0 years old and 60% were female. Of these children, 14 had mild asthma symptoms, five had moderate asthma symptoms, and six were diagnosed with severe asthma. There was only one child that had low levels of PA, seventeen children had moderate levels of PA, and seven children had high levels of PA.

The 17 participating parents were on average 43.4 ± 6.3 years old and 61% were female. Ten parents had a child with mild asthma, four parents had a child with moderate asthma, and three parents had a child with severe asthma. Thirteen parents had a child with moderate levels of PA, and four parents had a child with high levels of PA. No parents of children with low levels of PA participated.

The 21 healthcare providers that completed the study were on average 40.8 ± 11.3 years old and 85% were female. They had on average 15.9 ± 10.9 years of work experience in their profession and 7.9 ± 6.8 years of work experience with children with asthma.

### 3.2. Children’s Clusters

**Figure 3:**
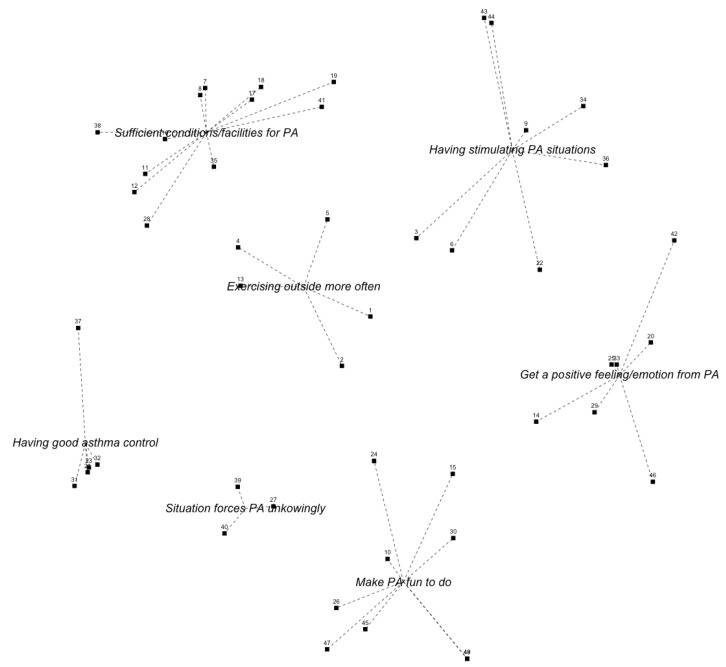
Concept map of the second group of children. The numbers of the ideas correspond to the ideas in Table 1.

In total, the four groups of children generated 51, 49, 25, and 36 ideas that were subsequently categorised into, respectively, ten, seven, four, and five clusters. Figure 3 shows an example of a concept map of one group of children. Table 1 shows the corresponding ideas to this concept map. The concept maps and lists of ideas of the other three groups of children can be found in Appendix A. Table 2 presents all clusters of the four groups of children together with their average importance rate and standard deviation (SD), and the label according to the PAD model.

The most important clusters for the four groups of children were getting a positive feeling of PA, having good asthma control, play outside more often, and get rewards for doing exercises or PA. Children in all groups mentioned that playing outside more often would stimulate their PA. Moreover, children in three out of four groups mentioned that making physical activities joyful or cool would stimulate them. Clusters regarding getting positive feelings of PA, being motivated by others, having access to sports facilities, and adding competitive elements to PA were mentioned by two out of four groups of children. Three out of four groups rated a cluster that was categorised as personal as most important, while one group rated an environmental cluster as most important.

### 3.3. Parents’ Clusters

Two groups of parents generated 37 and 36 ideas, which were grouped into, respectively, eight and five clusters. The concept maps and lists of ideas of both groups of parents can be found in Appendix A. Table 3 shows all clusters with their average importance rating and SD, and the label according to the PAD model. The most important clusters for the two groups of parents were about external factors that positively affect asthma control, and the child having sufficient knowledge about asthma, PA, and medication. Both these clusters were labelled as personal factors. There was only one overlapping cluster between both groups of parents, which regarded the child being in an environment that stimulates PA.

### 3.4. Healthcare Providers’ Clusters

The group of healthcare providers generated 116 ideas in total, which were grouped into thirteen clusters. The concept map and list of ideas of the healthcare providers can be found in Appendix A. Table 3 shows all final cluster names. Of all thirteen clusters, five were categorised as personal factors, and nine regarded environmental factors. The cluster named “child’s relatives have sufficient knowledge about asthma, PA, and medication” was rated as most important.

### 3.5. Combined Results of All Stakeholder Groups

**Figure 4:**
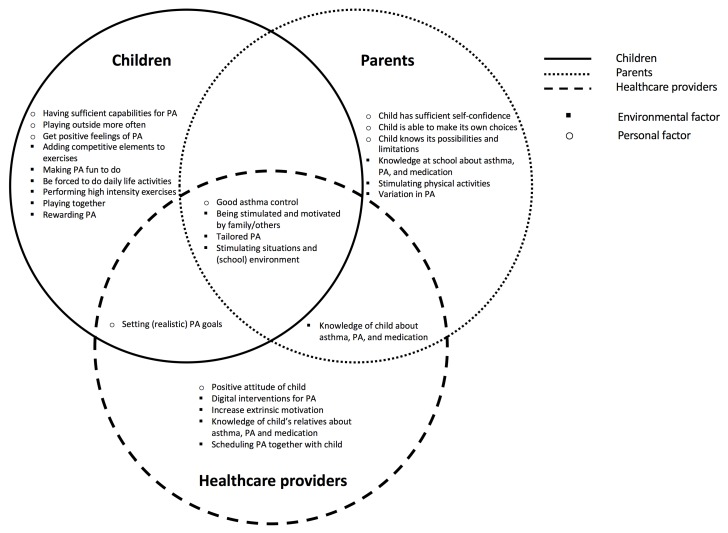
Venn diagram in which the clusters of all stakeholders are combined. The circles indicate the stakeholder groups with 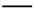 = children, 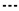 = parents, and 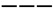 = healthcare providers. The symbols indicate the category of the cluster with: ○ = personal factor and ■ = environmental factor. The clusters are listed in random order.

Figure 4 shows the Venn diagram of all unique clusters generated by children, parents and healthcare providers. In total, nine clusters regarded personal factors, whereas seventeen clusters regarded environmental factors. Although most clusters were labelled as environmental factors, Table 2 and Table 3 show that five out of seven groups rated a cluster representing a personal factor as most important. The children identified nine unique clusters, i.e., that neither of the other stakeholder groups came up with. The parents and healthcare providers identified, respectively, six and five unique clusters. The cluster “setting (realistic) goals” overlapped for the children and healthcare providers, while the cluster that overlapped for the parents and healthcare providers was “knowledge of child about asthma, PA, and medication”. No overlapping cluster was found between the children and the parents. The four clusters “having good asthma control”, “physical activity and exercises that fit the child’s needs and capabilities”, “being stimulated and motivated by family/others”, and ’having stimulating situations and (school) environments” overlapped for all three stakeholder groups.

## 4. Discussion

### 4.1. Principal Results

This CM study explored children’s, parents’, and healthcare providers’ perspectives on factors promoting PA in children with asthma. According to all stakeholder groups, factors that promote PA in children with asthma are: having good asthma control, having physical activities and exercises that fit the children’s needs and capabilities, being stimulated and motivated by family members/others, and having stimulating situations and (school) environments.

The results of this study will be used to develop an intervention, for instance a smartphone application or a game, that helps healthcare providers to stimulate PA in children with asthma. Both the categories of the PAD model and the differences and similarities visualised in the Venn diagram will help to translate the promoting factors into requirements for the tool to be developed. Especially factors regarding intention, attitude, and self-efficacy are important for behavioural change. Moreover, we have special interest in factors that were mentioned by all stakeholder groups, since independently generating the same factors emphasises the value of these factors.

Fun, playing together, and being stimulated and motivated by family or others have been found to be important factors in stimulating PA levels of children with asthma. The factors fun and playing together were mentioned by the children, whereas the factor about stimulation and motivation from family or others were mentioned by all three stakeholders. The result regarding the factor fun was comparable to the results of Noonan et al. (2016) who showed that 10–11-year-old primary school children indicated the factors fun, enjoyment, competence, and PA provision as important for their (out-of-school) PA participation [22]. The factors regarding playing together and motivation and stimulation from family or others were not found for children with asthma specifically, but they were found in children with a disability [23].

Interestingly, both the healthcare providers and parents felt that children should know more about asthma and the effects of asthma on PA and medication to become more physically active, whereas this was not mentioned by the children themselves. When we noticed this discrepancy on the study days, we asked the children after completion of the CM session whether they felt that learning more about asthma, PA and medication would help them to become more physically active. The overall reaction was that they did not think so. In fact, many children indicated that they already knew everything about it. Their parents explained that the children indeed feel that they know a lot about it, but parents felt it was not enough, or that children were not able to convert their knowledge to corresponding behaviour. This was confirmed by results from a previous interview study among 15 children (7–17 years old) with asthma and their parents which showed that children do have knowledge about asthma-triggers, but need to be more aware of asthma symptoms and increase their knowledge about medication use [24]. In addition to the discrepancy between the children and the adults, a discrepancy between the parents and the healthcare providers was found. Although they both felt that increasing the children’s knowledge about asthma would stimulate PA, they did not feel the same about the knowledge of the children’s relatives regarding asthma. The healthcare providers felt that the children’s relatives, including the parents, should know more about asthma and the effect of asthma on PA and medication to stimulate the children’s PA, whereas the parents only felt that their children lack this knowledge.

Another interesting result was that no overlapping clusters were found between the children and their parents. There can be various reasons for this result. Parents may for instance have other insights in the disease and they may reflect on situations differently. On the other hand, we did find overlapping clusters between the children and the healthcare providers, so the age difference between the children and parents does not seem to be a reason for finding no overlapping clusters. The lack of overlapping factors may indicate that parents do not exactly know what will promote PA for their own child. Not knowing promoting factors of their own child might be a PA barrier on its own.

Buffart et al. (2009) also used the PAD model to assess stimulating factors of PA. This particular study assessed barriers and facilitators in young adults with a childhood-onset physical disability. They showed that PA related environmental facilitators in young adults with physical disabilities can be diverse while personal facilitators are limited to personal benefits and perceived rewards [25]. It therefore is not remarkable that our study resulted in more clusters with environmental factors than clusters with personal factors. Our results might suggest that environmental factors are more important for the stakeholders in promoting PA. On the contrary, five out of seven groups rating a personal factor cluster as most important might indicate that personal factors are more prominent in their ability to improve PA behaviour in children with asthma. Although the participants felt that solutions regarding their personal behaviour or skills might impact most on PA, they could think of more environmental solutions.

To the best of our knowledge, only one study assessed the influencing factors of PA in children with asthma [15]. Kornblit et al. (2008) examined the perceptions of barriers to PA and improvement opportunities in children with asthma. In contrast to our study, it was only about parental perceptions and the children’s perceptions were not studied. Although they focussed on the barriers, whereas we focussed on the facilitators, the results of both studies were comparable regarding the parents. Both the parents in the study of Kornblit et al. and the parents in our current study mentioned that children would be more physically active if schools would have more appropriate physical activities and a stimulating environment. Moreover, knowledge of asthma at schools was reported as important in both studies. Parents felt that their children would be more physically active if schools would know more about asthma symptoms in relation to PA, and when teachers would stimulate their children to take their asthma medications if necessary.

### 4.2. Strengths and Limitations

A strength of this study is the combination of qualitative and quantitative methods using CM. The CM method turned out to be a solid method to answer our research question. Moreover, the CM sessions were not only valuable to answer our research question, but also valuable for the children and parents. Many of them felt that they benefited from talking with fellow patients or fellow parents of a child with asthma.

Another strength is that we developed a tool to perform CM online, which enabled a number of healthcare providers to participate, while they would not have been able to participate in case of face-to-face sessions. The developed tool to perform CM online has promising possibilities for investigating other complex research questions or other participants. The generalisability of the results further strengthened our study. Most of our results are not typically asthma related, but show factors related to the patient’s environment and to coping strategies involving the patient itself, parents, teachers, and sports instructors. It is known that these factors apply for many other children with a chronic disease [26].

A limitation of the online CM procedure with healthcare providers was that a group discussion was not possible. In the construct of CM, a group brainstorm is advised to enable participants to, in this case, expend their thoughts about PA stimulation when receiving ideas from others [27]. We tried to minimise the effect of not having a group discussion by giving the participants the opportunity to add new ideas after reading the ideas of their fellow participants.

To minimise participant’s burden, the CM sessions were performed on one day. Therefore, the ideas of the different groups could not be combined. As a consequence, participants sorted and rated the ideas of their own group, resulting in one concept map for each group.

Another limitation of our study is the small number of participants per group and the imbalance between level of activity and asthma severity of children who participated. Although we tried to create a heterogeneous group of child participants, a relatively large part of the group had moderate levels of PA, while there was only one child with low levels of PA. Moreover, children with mild asthma symptoms were overrepresented compared to children with moderate or severe asthma. Similarly, parents of children with mild asthma and moderate levels of PA were overrepresented. The distribution of severity in asthma symptoms represents the real distribution among children with asthma, but the distribution of levels of activity did not. We gave information letters to many children with low levels of activity, but almost none of them were interested in participating. This might be due to the fact that children who are already physically active are more intrinsically motivated and therefore like to participate in a study that is about PA. Furthermore, our results might have been influenced by the skewed distribution of severity in asthma symptoms. It could be that participants with more severe asthma mention other promoting factors than children with mild asthma. On the other hand, all of our patients are treated by a paediatric pulmonologist in a specialised hospital instead of a general practitioner due to their severity in asthma symptoms. The homogeneous level of healthcare also might have influenced the promoting factors that were mentioned by the participants.

## 5. Conclusions

According to children with asthma, their parents, and healthcare providers, important factors supported by multiple stakeholder groups were having good asthma control, having access to PA facilities and tailored exercise, being stimulated and motivated by relatives, having PA stimulating situations and (school) environments, setting realistic goals, and having sufficient knowledge about asthma in relation to PA. These findings may inform future interventions and are a good start for setting the requirements of an intervention that helps healthcare providers in promoting children with asthma to become more physically active.

## Figures and Tables

**Table 1:** All ideas, together with their importance rates and standard deviation, of the first group of children categorised per cluster. The numbers of the ideas correspond to the numbers in the concept map shown in Figure 3.

Having good asthma control				Make PA fun to do		
32	If I am not ill	5.00 ± 0.00		48	If the exercise is new	5.00 ± 0.00
23	If I can breath properly	4.75 ± 0.50		24	If I could exercise together with other children	4.50 ± 1.00
21	If I am not short of breath	4.50 ± 0.58		30	If there is a game in which you have to exercise	4.50 ± 0.58
31	If I would not have asthma (symptoms)	4.00 ± 2.00		49	If I see someone else doing something new	4.50 ± 1.00
37	If I would be able to play in different places	3.00 ± 1.63		45	If I can learn someone else an exercise	4.00 ± 1.15
Sufficient conditions/facilities for PA				10	If there is music	3.50 ± 1.00
11	If I could run	4.50 ± 0.58		15	If I can show others what a joy being active is	3.25 ± 1.26
41	If I could jump on a trampoline	4.50 ± 1.00		47	If I see that someone else is exercising	3.00 ± 1.63
18	If I could bike	4.25 ± 0.96		26	If am distracted while exercising	2.25 ± 1.50
19	If there would be playground equipment	4.00 ± 0.82		Situation forces PA unknowingly		
28	If I would not have fear of heights	4.00 ± 2.00		40	If I don’t have to sit down	4.00 ± 2.00
38	If I could run in the forest	4.00 ± 1.15		27	If I am distracted while being physically active	3.50 ± 1.91
12	If I could walk	3.50 ± 0.58		39	If I’m not being active very often	1.00 ± 0.00
17	If I would go to school by bike	3.50 ± 1.00		Exercising outside more often		
7	If the weather is nice	3.25 ± 1.71		2	If I can do sports	4.25 ± 0.96
8	If it is not raining	3.25 ± 1.71		5	If II would go to play outside	4.00 ± 1.41
35	If I walk in a theme park or museum	2.75 ± 0.50		13	If I can show someone else what a joy playing outside is	3.50 ± 1.91
16	If I would not go to school by car	2.25 ± 1.50		1	If there are facilities in the neighbourhood to play outside	2.75 ± 1.50
Having stimulating PA situations				4	If I could climb in trees	2.25 ± 1.89
43	If we could play “boys against girls”	4.75 ± 0.50		Get a positive feeling/emotion from PA		
22	If I am alive	4.33 ± 1.15		29	If it is fun to do	5.00 ± 0.00
44	If it is a game	4.25 ± 0.96		25	If I am happy	4.75 ± 0.50
9	If I would participate in gym classes	3.25 ± 1.50		33	If PA is not boring	4.75 ± 0.50
3	If I would play soccer outside	3.00 ± 1.41		14	If I can go crazy	3.75 ± 0.96
6	If I am not home alone	3.00 ± 1.63		42	If it is exciting to do	3.50 ± 1.73
36	If there is a simulator for being in a theme park	2.25 ± 0.96		46	If I can imitate someone or something	3.50 ± 1.29
34	If there is a simulator for being outside	1.75 ± 0.96		20	If I am short of breath	2.25 ± 1.26

**Table 2:** Final clusters for promoting PA of each group of children. Ordered on importance score and categorised with the use of the PAD model. White cells are personal factors with: ● = intention, ♣ = attitude, ■ = self-efficacy, ★ = health condition, and ◆ = personal barriers and facilitators. Grey cells are environmental factors with ▲ = social influence, and ✻ = environmental barriers and facilitators.

Children Group 1		Children Group 2		Children Group 3		Children Group 4	
●	Get a positive feeling of PA 3.9 ± 0.4	★	Having good asthma control 4.3 ± 0.8	●	Play outside more often 3.2 ± 0.5	✻	Get rewards for doing exercises or PA 3.9 ± 0.4
▲	Playing together 3.7 ± 0.3	♣	Get positive feeling and emotion from PA 3.9 ± 1.0	✻	Have cool and special activities 3.1 ± 0.6	✻	Tailored PA 3.8 ± 0.5
▲	Be motivated by others 3.7 ± 0.6	✻	Make PA fun to do 3.8 ± 0.9	▲	Being motivated by others to exercise 2.8 ± 0.7	✻	Make exercises joyful 3.7 ± 0.3
◆	Sufficient capabilities for PA 3.6 ± 0.2	✻	Sufficient conditions and facilities for PA 3.6 ± 0.7	✻	Add competitive elements to PA 2.4 ± 0.1	●	Play outside more often 3.7 ± 0.6
✻	Have access to nice sports and exercises 3.6 ± 0.7	●	Exercising outside more often 3.4 ± 0.8			●	Set exercise goals 2.9 ± 0.3
●	Perform high intensity exercises 3.1 ± 0.8	✻	Having stimulating PA situations 3.3 ± 1.1				
✻	Add competitive elements to PA 2.9 ± 0.8	✻	Situation forces PA unknowingly 2.8 ± 1.6				
✻	Have joyful activities to perform 2.9 ± 0.6						
●	Play outside more often 2.4 ± 0.7						
✻	Be forced to do daily life activities 1.6 ± 0.6						

**Table 3:** Final clusters for promoting PA of each group of parents and healthcare providers. Ordered on importance score and categorised with the use of the PAD model. White cells are personal factors with: ● = intention, ♣ = attitude, ■ = self-efficacy, ★ = health condition, and ◆ = personal barriers and facilitators. Grey cells are environmental factors with ▲ = social influence, and ✻ = environmental barriers and facilitators.

Healthcare Providers Group 1		Parents Group 1		Parents Group 2	
✻	Child’s relatives have sufficient knowledge about asthma, PA, and medication 4.2 ± 0.1	◆	Child has sufficient knowledge about asthma, PA, and medication 3.7 ± 0.6	★	Good asthma control despite triggers 3.9 ± 0.3
▲	Parents that support and stimulate PA 4.2 ± 0.8	✻	Tailored PA 3.6 ± 0.4	✻	School environment and policy stimulate PA 3.9 ± 0.3
✻	Having stimulating preconditions for PA 4.1 ± 0.1	✻	Variation in physical activities 3.6 ± 0.5	▲	Exemplary behaviour of relatives regarding PA 3.8 ± 0.4
◆	Child has sufficient knowledge about asthma, PA, and medication 4.1 ± 0.4	■	Child has sufficient self-confidence 3.6 ± 0.7	■	Child is able to make its own choices 3.6 ± 0.7
★	Child reaches good asthma control 4.1 ± 0.4	▲	Having family that stimulates PA 3.5 ± 0.5	✻	Child is in environment that stimulates PA 3.5 ± 0.5
✻	Environmental conditions that stimulate PA 4.0 ± 0.4	✻	School has sufficient knowledge about asthma, PA, and medication 3.4 ± 0.1		
✻	Having digital interventions that stimulate PA 3.9 ± 0.4	✻	Child is in environment that stimulates PA 3.3 ± 0.1		
✻	Increasing extrinsic motivation 3.8 ± 0.6	■	Child knows its possibilities and limitations 3.3 ± 0.6		
♣	Child is, acts and thinks positive 3.8 ± 0.7				
▲	Organise PF in daily schedule for child 3.7 ± 0.6				
✻	Having accessible PA locations/equipment 3.6 ± 0.8				
✻	Tailored PA 3.5 ± 0.6				
●	Setting realistic goals 3.5 ± 0.8				

**Figure A1:**
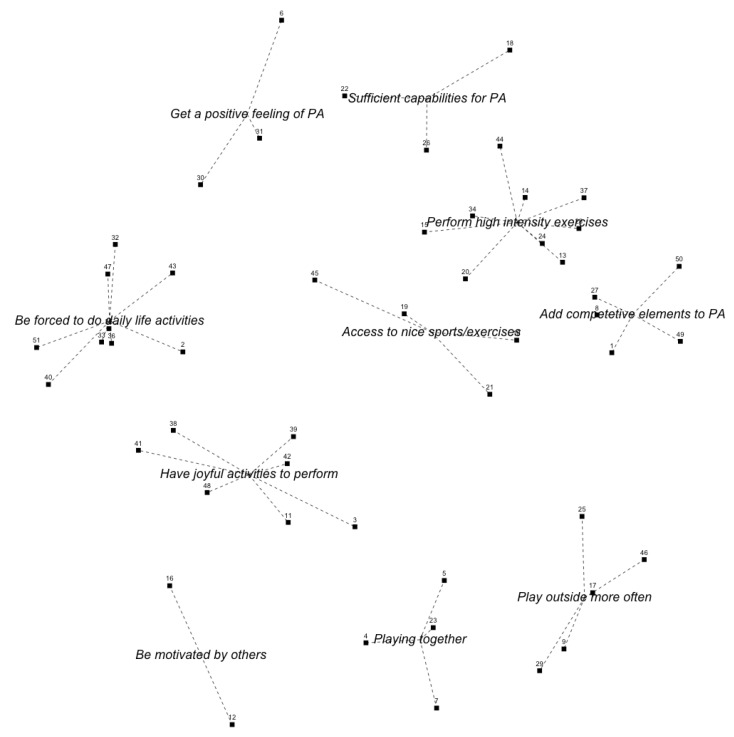
Concept map of the first group of children. The numbers of the ideas correspond to the ideas in Table A1.

**Figure A2:**
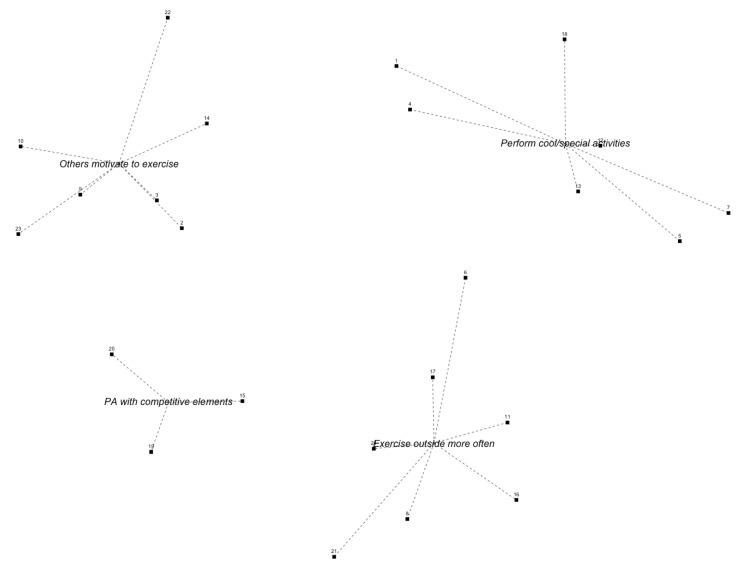
Concept map of the third group of children. The numbers of the ideas correspond to the ideas in Table A2.

**Figure A3:**
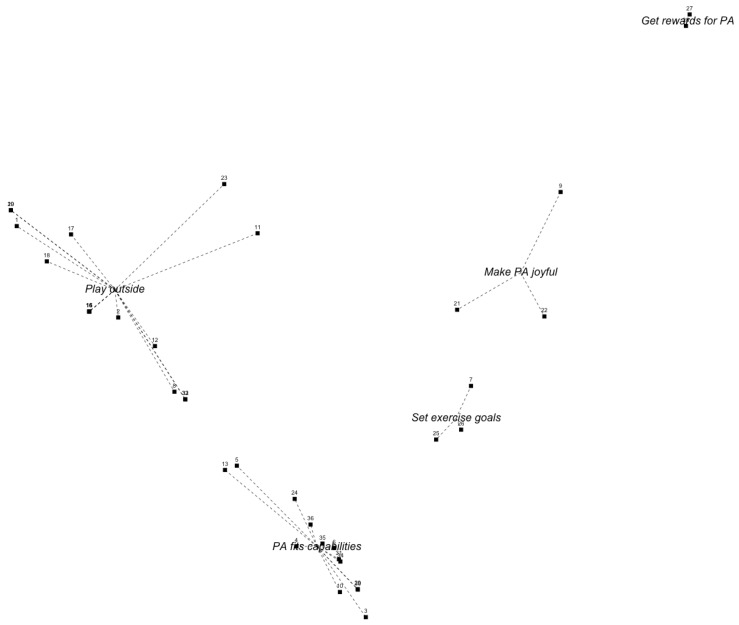
Concept map of the fourth group of children. The numbers of the ideas correspond to the ideas in Table A3.

**Figure A4:**
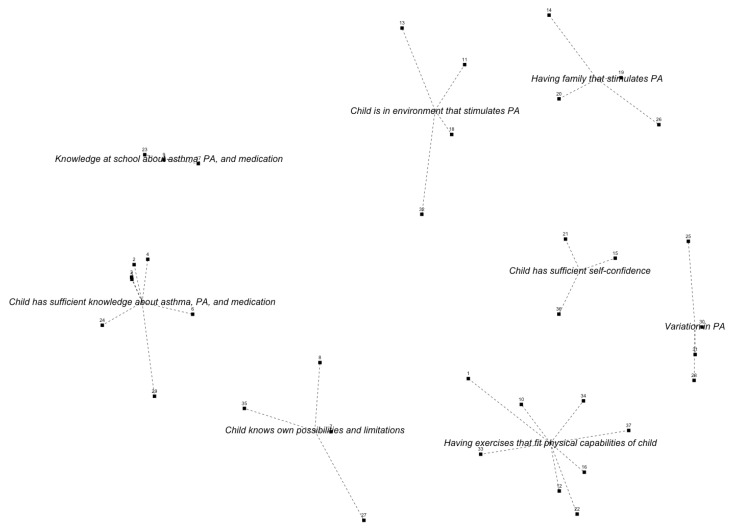
Concept map of the first group of parents. The numbers of the ideas correspond to the ideas in Table A4.

**Figure A5:**
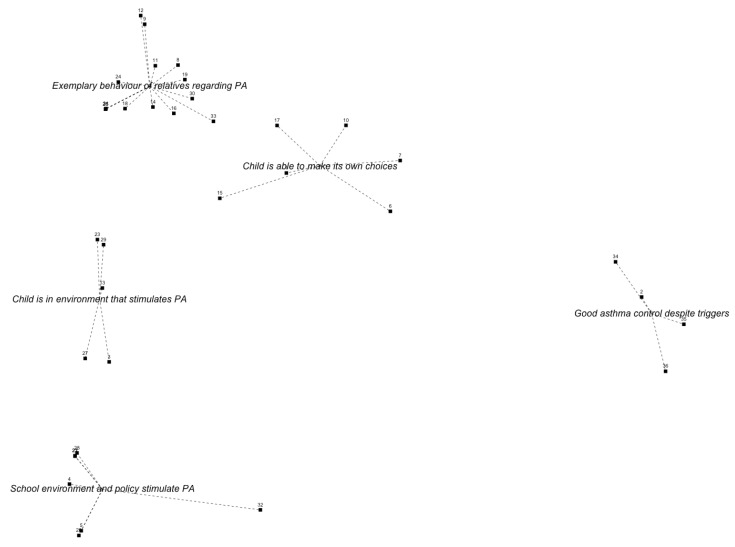
Concept map of the second group of parents. The numbers of the ideas correspond to the ideas in Table A5.

**Figure A6:**
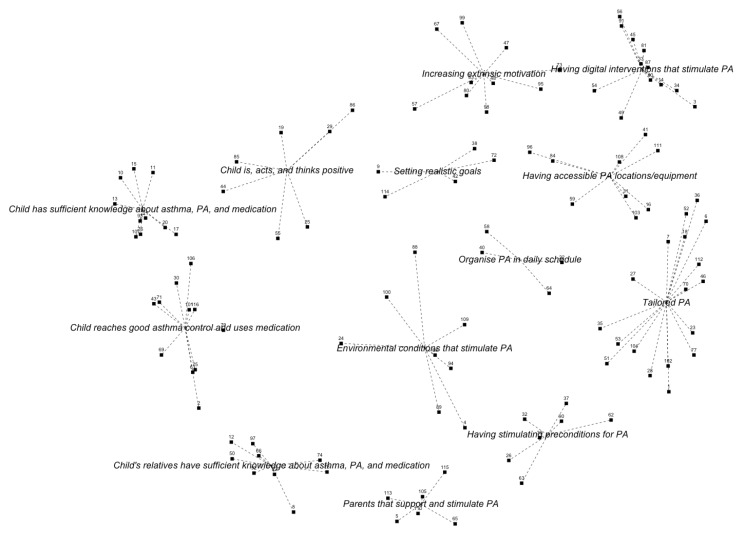
Concept map of the group of healthcare providers. The numbers of the ideas correspond to the ideas in Table A6.

## Abbreviations

The following abbreviations are used in this manuscript:

CM	Concept Mapping
MSD	Multi Dimensional Scaling
PA	Physical Activity
PAD	Physical Activity for people with a Disability
SD	Standard Deviation

## Appendix A. Final Concept Maps and Ideas of All Groups

### Appendix A.1. Children Group 1

**Table A1:** All ideas, together with their importance rates and standard deviation, of the first group of children categorised per cluster. The numbers of the ideas correspond to the numbers in the concept map shown in Figure A1.

Be forced to do daily life activities				Have joyful activities to perform		
2	If I would drink and eat a lot	2.56 ± 1.01		3	When you do something you like	4.33 ± 0.87
32	If I can walk somewhere	2.56 ± 1.59		11	When you like something	4.11 ± 1.36
43	If I would think	2.00 ± 1.41		48	If you would hear a sound that supports you to move more	2.89 ± 1.45
40	If I had to inflate the tires of my bike	1.89 ± 1.05		42	If I would play with construction materials	2.78 ± 1.09
33	If I would get up from the chair	1.22 ± 0.44		38	If I could demolish things	2.56 ± 1.01
51	If I would walk to the bathroom	1.22 ± 0.44		41	If I would talk about exercising	2.11 ± 0.78
36	If I would turn on a light	1.11 ± 0.33		39	If I can sing	1.78 ± 1.3
35	If I would fall	1.00 ± 0.00		Sufficient capabilities for PA		
47	If I would get something to drink	1.00 ± 0.00		26	If I would take my inhalation medication	3.78 ± 1.48
Playing outside more often				22	If I have self-confidence	3.56 ± 1.48
9	If you make a game of it	3.56 ± 0.88		18	If you receive certificates or medals	3.44 ± 1.51
17	If I can pay outside a lot	2.14 ± 1.46		Be motivated by others		
25	If I can swing	2.11 ± 0.60		12	If you get support from your parents and friends	4.11 ± 1.05
46	If I would play “man overboard”	1.54 ± 1.00		16	If I look at others to see how they are doing it	3.33 ± 1.12
29	If I would play “Pokemon Go”	2.00 ± 1.05		Get a positive feeling of PA		
Have access to nice sports and exercises				30	If I stay healthy after being active	4.33 ± 0.71
10	If you could do sports that you enjoy	4.44 ± 0.73		6	If I would receive points and I would be rewarded for being active is	3.89 ± 0.93
45	If your whole body starts to move	4.00 ± 1.32		31	If I would be brave enough to do so	3.44 ± 1.33
19	If you can dance	3.25 ± 1.58		Playing together		
21	If you can go skiing	2.89 ± 1.05		4	If I do it together with my parents	4.22 ± 0.67
Perform high intensity exercises				5	If I go to “Jumping Jack” with my friends	3.67 ± 1.22
14	If you can play sports	4.33 ± 1.00		7	If I am together with my friends	3.57 ± 1.40
34	If I would have gym classes at school more often	3.78 ± 0.67		23	If I can play with my friend	3.50 ± 0.76
15	If I can run	3.56 ± 0.88		Add competitive elements to PA		
37	If I would combine different types of sports	3.56 ± 0.88		8	If I would swim	4.11 ± 0.93
20	If I can bike	3.11 ± 1.45		27	If I could swim at a competition	3.00 ± 1.22
28	If I can play water polo	2.67 ± 1.50		1	If I can kick a ball	2.67 ± 1.22
44	If I would not sport too much	2.44 ± 1.01		50	If I can play soccer outside	2.67 ± 1.80
24	If I can do freerunning	2.25 ± 1.04		49	If I can play “curbs”	1.89 ± 1.05
13	If I can be the goalkeeper	2.22 ± 1.20				

### Appendix A.2. Children Group 3

**Table A2:** All ideas, together with their importance rates and standard deviation, of the third group of children categorised per cluster. The numbers of the ideas correspond to the numbers in the concept map shown in Figure A2.

Being motivated by others to exercise				Play outside more often		
22	When there would be more gym classes at school	4.00 ± 1.15		8	If you would play active outdoor games	3.86 ± 1.46
23	If you receive an reward when you are active	3.29 ± 1.50		6	If children with asthma would always use a bike to go somewhere	3.57 ± 1.51
9	If someone motivates a child to win	2.86 ± 1.86		21	If there were nice walking routes for children	3.29 ± 1.11
3	If children with asthma still try to do something that they are excited about	2.71 ± 1.11		11	If a child with asthma would play in an indoor playground	3.14 ± 1.35
14	If there is a build-up training schedule	2.71 ± 1.38		24	If the weather would be better more often	3.00 ± 1.15
10	If children with asthma are paying attention to what they do and whether they can sustain it	2.14 ± 1.07		17	If scouting activities are offered more often by scouting clubs	2.86 ± 1.21
2	Prove that you can do so in case others think that you cannot	2.00 ± 1.26		16	If you can play a nice outdoor game with your friends	2.43 ± 1.51
Perform cool and special activities				Add competitive elements to PA		
12	If a child with asthma would go “disco swimming”	4.00 ± 1.00		15	If you would talk with other children more often	2.57 ± 1.51
5	If a child with asthma would play Just Dance more often	3.43 ± 1.13		20	If you need to reach a specific distance in a specific time frame	2.43 ± 1.13
7	When you create a game with a motion sensor	3.43 ± 1.40		19	If you occasionally have to run to school	2.29 ± 1.25
13	If a child would go to a “trampoline park”	3.43 ± 1.51				
18	If you would perform dancing at a dance centre	2.67 ± 1.37				
1	If a child with asthma would jump rope more often	2.43 ± 0.98				
4	If a child with asthma would skydive more often	2.43 ± 1.51				

### Appendix A.3. Children Group 4

**Table A3:** All ideas, together with their importance rates and standard deviation, of the fourth group of children categorised per cluster. The numbers of the ideas correspond to the numbers in the concept map shown in Figure A3.

Set exercise goals				Make exercises joyful		
25	If a child with asthma has an activity goal	3.40 ± 1.34		22	If there were film clips for children with asthma with “weird” movements to imitate	4.00 ± 1.73
26	If you choose an activity that you can do well in order to win	3.20 ± 1.79		9	If the activity is being rewarded with something nice	3.60 ± 0.55
7	If children with asthma would dance in order to deal with their emotions	2.20 ± 1.64		21	If a child with asthma would do “The Netherlands in motion” with their grandmother	3.40 ± 1.82
Get rewards for doing exercises or PA				Play outside more often		
28	If children receive rewards for working hard	4.20 ± 0.45		32	If we play “trefbal” more often in gym classes at school so that you are very active	4.60 ± 0.55
27	If children will feel happy after doing working hard	3.60 ± 1.14		8	If gym classes are fun and challenging so that you can always actively participate	4.40 ± 0.89
Tailored PA				17	If a child with asthma would more often go to school by bike	4.20 ± 0.84
30	If children with asthma would distribute their energy during sports activities in order to sustain	4.60 ± 0.55		15	If you can be active together with your friends and play a match	4.00 ± 0.71
5	If a child with asthma is replaced in time and/or more frequently during a soccer game	4.20 ± 0.84		14	If children with asthma would play outside, especially if they are bored	4.00 ± 1.00
6	If children with asthma would dance more often in order to train their condition	4.20 ± 0.84		33	If I can play “trefbal” with many balls	4.00 ± 1.00
29	If children with asthma, who suffer a lot, would do sports that they can maintain well	4.20 ± 0.84		16	If a child with asthma more often plays active outdoor games	3.80 ± 1.30
34	If you would dance with crazy moves	4.20 ± 0.84		12	If a child with asthma would go to the swimming pool by bike	3.75 ± 0.50
36	If a child with asthma could do horse riding	4.20 ± 1.79		1	If a child with asthma would play outside more often	3.60 ± 1.14
13	If a child with asthma would run every day	4.00 ± 1.41		23	If a child with asthma would go roller skating together with its family	3.60 ± 1.14
10	If children with asthma would do sports that they really like to do	3.80 ± 1.10		19	If your would jump rope at school in breaks	3.20 ± 0.45
3	If children with asthma would participate more often in less intensive sports	3.60 ± 1.14		20	If you would use the break at school better	3.00 ± 1.58
4	If children with asthma join sports clubs so that they are expected to join the training	3.60 ± 1.95		18	If you can play tag more often so that you can get tired together with other children	3.00 ± 1.87
35	When there are more often funny games during the warming up	3.40 ± 1.34		11	When the parents of a child with asthma would not have a car	3.00 ± 1.87
24	If there would be a special asthma team sport in which it is allowed to replace players frequently	3.00 ± 1.41		2	If a child with asthma would more often do bicycle matches with other children	2.80 ± 0.84
31	If sports have a steady increase in activities and intensity	2.80 ± 1.79				

### Appendix A.4. Parents Group 1

**Table A4:** All ideas, together with their importance rates and standard deviation, of the first group of parents categorised per cluster. The numbers of the ideas correspond to the numbers in the concept map shown in Figure A4.

Tailored PA				Child knows its possibilities and limitations		
34	If a child with asthma experiences success more often	4.25 ± 0.87		8	If a child knows its limits	3.92 ± 1.00
1	If a child has confidence that the exercise will not lead to an asthma attack	4.25 ± 0.97		7	Find out together achievability in terms of movement	3.75 ± 0.87
16	If a child can do the same exercises as other children	3.67 ± 1.15		35	Find out together achievability in terms of allergy	2.83 ± 1.40
37	If there is an element of competition in the activity	3.58 ± 1.31		27	If child with asthma does not use asthma as an excuse	2.75 ± 1.48
22	If an activity is accessible and easy to go to	3.50 ± 1.17		Child has sufficient knowledge about asthma, PA, and medication		
33	If a child with asthma can keep his physical fitness level stable	3.50 ± 1.31		2	Correct use of medication	4.50 ± 0.90
10	If a child with asthma likes the activity just as much as children without asthma	3.33 ± 1.44		3	If a child with asthma uses the right dose of medication	4.42 ± 1.08
12	Meeting sports heroes that also have asthma	2.92 ± 1.56		6	Knowing that a normal breathing pattern is	3.75 ± 1.36
Having family that stimulates PA				4	If a child with asthma knows what asthma is	3.50 ± 1.09
14	If children have enough time and possibilities to exercise at home	3.92 ± 1.16		24	When there would be an action plan and the child knows how to use it	3.25 ± 1.48
19	If parents exercise together with the child	3.67 ± 0.89		5	If a child with asthma knows the effect of the medication	3.17 ± 1.40
20	If parents watch the exercising child	3.50 ± 1.45		29	If a child with asthma always carries its medication with him/her	3.08 ± 1.38
26	If screen time is reduced	2.83 ± 1.27		School has sufficient knowledge about asthma, PA, and medication		
Variation in physical activities				9	The school environment of a child with asthma knows what asthma is	3.50 ± 1.17
30	If there is variability in activities	4.33 ± 0.78		17	If a school knows more about asthma	3.42 ± 0.79
31	If there is a moment of rest within an activity	3.42 ± 1.124		23	If there are agreements with teachers about exercise and medication of the child	3.33 ± 1.07
25	If exercising is being rewarded	3.33 ± 1.30		Child is in environment that stimulates PA		
28	If active and passive periods are alternated	3.33 ± 1.30		13	If children have enough time and space to exercise at school	3.42 ± 1.07
Child has sufficient self-confidence				11	If others (parents, friends, school, therapists) motivate the child	3.33 ± 1.37
21	If a child with asthma has enough self-esteem to dare to move	4.08 ± 0.79		32	if a healthcare provider emphasises exercise and sports positively	3.25 ± 1.29
15	If children do things that they like	4.00 ± 1.04		32	If a child with asthma is stimulated to keep being active during a period of illness	3.25 ± 1.42
36	Meeting a hero who also has asthma	2.75 ± 1.54				

### Appendix A.5. Parents Group 2

**Table A5:** All ideas, together with their importance rates and standard deviation, of the second group of parents categorised per cluster. The numbers of the ideas correspond to the numbers in the concept map shown in Figure A5.

Child is in environment that stimulates PA				Good asthma control despite triggers		
23	If there were more possibilities for indoor activities focussed on PA	4.00 ± 0.00		2	When there is asthma control	4.20 ± 1.30
3	If the child is guided well during sports performances	3.60 ± 0.55		35	If the air would be cleaner	4.00 ± 1.22
29	If there is a structure in weekly exercises	3.60 ± 0.89		36	If smoking near children would be forbidden	4.00 ± 1.41
13	If PA is facilitated by the environment	3.60 ± 1.14		34	When the side effects of asthma medication would be less	3.40 ± 1.52
27	When a child has to sit, it can sit on a gym ball	2.60 ± 0.89		Exemplary behaviour of relatives regarding PA		
School environment and policy stimulate PA				25	If parents would set a good example regarding sports is	4.40 ± 0.89
21	If more exercise materials would be offered at school during the break	4.60 ± 0.55		30	If a child exercises together with other people	4.40 ± 1.34
20	When gym classes at school are offered at least twice a week	4.20 ± 0.45		24	If parents would set a good example regarding screen time	4.20 ± 1.10
22	If teachers stimulate PA during the break	4.20 ± 0.84		9	If a child plays an active digital game	4.00 ± 1.22
28	If activities at school are really done instead of shown in a screen	4.00 ± 0.00		12	If exercising is an implied condition to play a game	4.00 ± 1.22
5	If there is sufficient variance during gym classes	3.80 ± 1.10		14	If a hobby is also related to physical activity	3.80 ± 0.45
4	During school lessons being motivated to be active	3.40 ± 0.89		26	If other help children in deciding what to do	3.80 ± 0.45
32	If phones are forbidden at schools	3.40 ± 2.19		18	If the child is being rewarded	3.80 ± 0.45
Child is able to make its own choices				8	If there is not too much distraction because of screens	3.80 ± 1.64
1	If they can perform sports that they really like	4.80 ± 0.45		33	If a child wants to join a group that exercises a lot	3.60 ± 0.89
17	If the child understands how many activities he/she has performed	4.00 ± 0.71		31	If a child performs active tasks	3.60 ± 1.14
10	If children accept each other’s physical problems and children are not excluded	3.60 ± 0.89		11	If there are games in which children with asthma can participate at different intensity levels	3.40 ± 0.55
6	If the child starts to use medication when needed	3.40 ± 1.34		19	If physical activity includes elements of competition	3.40 ± 0.55
7	If the child is not ashamed of using medication during sports	3.00 ± 0.71		16	If not only sports are labelled as physical activity	3.20 ± 1.30
15	If a child does not sit all the time	3.00 ± 0.71				

### Appendix A.6. Healthcare Providers

**Table A6:** All ideas, together with their importance rates and standard deviation, of the group of healthcare providers categorised per cluster. The numbers of the ideas correspond to the numbers in the concept map shown in Figure A6.

Child is, acts, and thinks positive				Having accessible PA locations/equipment		
44	Show children what they can do themselves to be less short of breath	4.48 ± 0.98		96	If enjoyment of PA is increased	4.29 ± 0.78
29	If PA experiences are positive and without association of pain and dyspnea	4.43 ± 0.60		84	If exercises are offered structurally or children get a reminder	4.19 ± 0.75
86	If there is a good balance between exercise and relaxation	4.29 ± 0.72		103	If the exercise can be done with either good weather or bad weather	4.14 ± 0.65
85	If they know and respect their physical limits well	4.10 ± 0.94		41	If children can invite friends to participate in a game or sports program	4.05 ± 0.92
55	If the child has a good physical capacity	3.62 ± 0.92		16	If sports programs are in line with the trends of the moment	3.76 ± 0.94
19	If they get example of famous Dutch people having asthma and playing sports too	2.90 ± 0.77		59	If activities are offered as alternatives for watching television	3.67 ± 0.80
25	If the paediatrician gives them a book with ideas and tips about physical activity	2.86 ± 0.96		108	If periods of watching television are alternated with PA and this is rewarded	3.14 ± 1.20
Having digital interventions that stimulate PA				31	If a child would walk instead of bike to be active for a longer time	2.76 ± 1.14
49	If exercise is made joyful with use of a game	4.48 ± 0.60		111	If the child would receive a cheap treadmill with a game to walk at home	2.10 ± 1.14
14	If there is an app or game that can be used indoors and stimulates exercise	4.29 ± 0.64		Parents that support and stimulate PA		
56	If there is an app that monitors PA and rewards PA in a nice way	4.24 ± 0.83		105	If parents would also be enthusiastic about physical activity	4.71 ± 0.46
60	If there is an exercise game that they can play together with other children to stimulate each other	4.19 ± 0.75		113	If parents would show that they are proud of their children	4.67 ± 0.48
54	If exercising would be challenging	4.10 ± 0.77		110	If parents support the child to be physically active and are a good example	4.62 ± 0.59
3	If there is a rewarding system for PA	4.05 ± 0.97		115	If parents would participate in interventions with different goals	4.29 ± 0.78
91	If there is a (computer) game where you can collect points and achieve higher levels when being active	4.00 ± 0.89		5	If parents would not be too protective is	3.95 ± 0.92
87	When exercises are delivered through different media (tablet, phone, activity tracker)	4.00 ± 0.89		65	If parent positively support children during sports games instead of shouting	2.71 ± 1.27
45	If there is a smartphone app that links PA behaviour to peers with competition elements	3.76 ± 0.89		Tailored PA		
81	When there would be different exercise apps with different themes	3.62 ± 0.86		77	If the school yard is a challenging environment to exercise	4.38 ± 0.59
34	If there are inspiring outdoor tasks. For instance with google maps and taking pictures	3.62 ± 1.02		27	If tailored PA is offered	4.05 ± 0.92
33	If there exist virtual reality exercise games	2.95 ± 1.24		53	If children walk/bike to school instead of going by car	4.05 ± 1.02
Increasing extrinsic motivation				104	If it is possible to do the exercise inside instead of outside in winter times	4.05 ± 0.67
98	If they gain success experiences	4.71 ± 0.46		18	If there is a group training instead of an individual training	3.81 ± 0.81
48	If the exercises correspond to the child’s strengths	4.43 ± 0.60		70	If they have the opportunity to try out different sports	3.76 ± 0.89
95	If they can determine what they want to do in terms of PA	4.14 ± 0.85		6	If exciting materials are offered for all ages	3.71 ± 0.90
47	If exercise is addictive: being active is rewarded and being inactive is not	4.00 ± 0.84		23	If there is variation in sports options	3.71 ± 0.78
99	If there exists an app for children with asthma with useful tips and tricks	4.00 ± 1.14		51	If children with asthma are matched to those who are very active	3.57 ± 1.03
73	If they are rewarded for good PA behaviour	3.95 ± 1.02		35	If the difference with peers in physical capacity is not that big	3.52 ± 0.81
80	If exercising results in measurable results like physical fitnett or increased muscle strength	3.57 ± 1.08		36	If they exercise together with other children with asthma	3.38 ± 1.12
57	If they have contact with other children suffering from asthma	3.52 ± 0.81		46	If they exercise together with other children with a chronic disease	3.38 ± 1.12
83	If the child is offered a questionnaire to help determining what he/she likes	3.29 ± 1.10		102	If there is open access to sports facilities	3.24 ± 1.14
67	If the child watches recordings of his own activities to see what he/she did	2.81 ± 1.08		112	If they temporarily exercise in a group with lifestyle interventions	3.14 ± 0.91
Child reaches good asthma control and uses medication				28	If a sports day is organised for children with asthma	2.76 ± 1.22
101	If the correct medication and dosage are used before/during sports	4.67 ± 0.58		52	If exercise materials can be borrowed from friends	2.71 ± 1.06
61	If asthma control is reached	4.43 ± 0.68		1	If presence at sports is rewarded with membership awards	2.71 ± 1.27
43	If the correct medication is used and there is good medication adherence	4.38 ± 0.92		7	If daily life activities are combined with exercises	2.43 ± 0.98
69	If the child, parents, and teachers know about the use of bronchodilators	4.38 ± 0.97		Organise PA in daily schedule for child		
71	If the child always carries its medication with him/her	4.29 ± 1.01		58	If people see the strengths of a child	3.33 ± 1.12
106	If fear for an asthma attack is reduced (both for the child and its relatives)	4.29 ± 1.06		22	If there exists apps in which family is involved to play against each other	4.00 ± 0.84
30	If it is clear that the child with asthma needs to be as physically active as its healthy peers	4.19 ± 0.93		64	If they join an exercise program like Fitkids	3.29 ± 1.15
79	If it is not stupid to take medication	4.05 ± 1.07		40	If an exercise period is planned	3.05 ±1.32
116	If medication is easy to take with you	4.00 ± 1.10		Having stimulating preconditions for PA		
75	If an irritated nose is treated	3.48 ± 1.25		32	If parents exercise with together with children to stimulate PA	4.19 ± 0.87
2	If children with asthma are not overweighted too	3.14 ± 1.35		26	If there would be financial support for families with financial problems	4.14 ± 0.79
Chid has sufficient knowledge about asthma, PA, and medication				37	If there is a living environment in which they can play	4.14 ± 0.91
107	If the child has enough knowledge about the disease	4.76 ± 0.44		39	If the environment is safe for exercising	4.10 ± 0.70
76	If the child knows that exercising does not result in physical damage	4.52 ± 0.60		62	If there are more gym classes at school	4.10 ± 1.18
15	If a child knows the difference between shortness of breath and dyspnea	4.38 ± 0.97		90	If there would be enough sports facilities in their neighbourhood	4.00 ± 0.63
10	If a child knows the difference between feeling short of breath and being short of breath	4.33 ± 1.00		63	If being different is accepted as such	3.90 ± 1.04
93	If a child does not have to be dyspneic after exercising	4.19 ± 1.21		Setting realistic goals		
20	If the importance of physical activity is clear	4.05 ± 0.74		9	Setting realistic goals	4.24 ± 1.00
21	If the child knows about physical capacity and having less energy during an exacerbation	4.00 ± 1.00		114	Setting realistic together with and for the child	4.14 ± 1.01
11	If the child is aware of the importance of a good physical fitness	3.90 ± 0.77		42	If exercising has a purpose other than health benefits	3.43 ± 0.98
13	If it is clear that, there could be other reasons, in addition to asthma, why the child feels short of breath	3.90 ± 1.14		72	If the exercise is not too hard to perform	3.33 ± 1.06
17	If the child knows how to perform a warming up and cooling down correctly	3.29 ± 1.23		38	If gross and fine motor skills are alternated	2.38 ± 0.92
Chid’s relatives have sufficient knowledge about asthma, PA, and medication				Environmental conditions that stimulate PA		
8	If the child is less exposed to smoke	4.38 ± 1.12		68	If playing outside would be stimulated	4.48 ± 0.60
74	If sports teachers and trainers help the child when it experiences asthma symptoms	4.29 ± 0.78		94	If time is made for PA	4.33 ± 0.73
66	If sports teachers know more about the “do’s and don’ts” in children with asthma	4.29 ± 0.85		100	If the child can gain PA experiences from an early age	3.50 ± 0.76
97	If there is sufficient knowledge among sports teachers and clubs about the impact of asthma on a child	4.29 ± 0.90		4	If everyone stimulated a child with asthma to participate in activities	4.29 ± 0.72
92	If there is information for the parents about PA, physical fitness, and psychosocial development	4.24 ± 0.70		89	If the child has a good bicycle to go to school	3.76 ± 1.09
82	If parents would be less afraid that the child could get an asthma attack during physical activity	4.24 ± 0.77		88	If a week schedule is made with several options per day	3.67 ± 1.06
50	If school is well informed that the child could get an asthma attack during physical activity	4.24 ± 0.77		24	If screen time is limited	3.62 ± 1.02
12	If there is information about asthma and the impact on a child for all cultures	4.05 ± 0.80		109	If physiotherapists help inactive children in choosing sports	3.29 ± 1.19
78	If peers and adults show that they understand the complaints of a child	4.05 ± 0.92				
